# Importance of old bulls: leaders and followers in collective movements of all-male groups in African savannah elephants (*Loxodonta africana*)

**DOI:** 10.1038/s41598-020-70682-y

**Published:** 2020-09-03

**Authors:** Connie R. B. Allen, Lauren J. N. Brent, Thatayaone Motsentwa, Michael N. Weiss, Darren P. Croft

**Affiliations:** 1 Centre for Research in Animal Behaviour, College of Life and Environmental Sciences, University of Exeter, Exeter, EX4 4QG UK; 2 Elephants for Africa, 5 Balfour Road, London, N5 2HB UK; 3 Elephants for Africa, Mailbox 148 HAK, Maun, Botswana

**Keywords:** Animal behaviour, Zoology, Ecology, Behavioural ecology, Conservation biology

## Abstract

In long-lived social species, older individuals can provide fitness benefits to their groupmates through the imparting of ecological knowledge. Research in this area has largely focused on females in matrilineal societies where, for example, older female African savannah elephants (*Loxodonta africana*) are most effective at making decisions crucial to herd survival, and old post-reproductive female resident killer whales (*Orcinus orca*) lead collective movements in hunting grounds. In contrast, little is known about the role of older males as leaders in long-lived social species. By analysing leadership patterns of all-male African savannah elephant traveling groups along elephant pathways in Makgadikgadi Pans National Park, Botswana, we found that the oldest males were more likely to lead collective movements. Our results challenge the assumption that older male elephants are redundant in the population and raise concerns over the biased removal of old bulls that currently occurs in both legal trophy hunting and illegal poaching. Selective harvesting of older males could have detrimental effects on the wider elephant society through loss of leaders crucial to younger male navigation in unknown, risky environments.

## Introduction

During coordinated group movements certain individuals can consistently arise as “leaders” with a regular influence over group decisions, with high dominance rank^1^, bold temperament^2^ and greater age (often associated with enhanced knowledge or experience^3^) noted as common traits characterising leaders (for review, see^4^). Whilst in some cases leadership can be a passive process, a consequence of simple consensus decisions to maintain group cohesion ^5^, in other cases leaders actively communicate their intent to recruit followers^6^. In long-lived species, older individuals often respond more appropriately to complex, changing environments^7,8^, providing substantial fitness benefits to younger group mates. For example, older matriarchs are more effective at mobilising groups in response to predation threats and conspecifics in female African savannah elephants (*Loxodonta africana*)^8,9^, Similarly, in resident killer whale (*Orcinus orca*) groups there is a greater reliance on older, post-menopausal females as leaders of hunting groups in years of low salmon abundance^10^.

Non-human research in this area has tended to focus on females and less attention has been given to the potential for old males to act as repositories of ecological knowledge and leaders in long-lived social species. In many social mammals, males are often assumed to be replaceable because they are typically the dispersing sex^11^, and old males may be reproductively redundant, which is commonly used as an argument to support the legal trophy hunting of old males in many species^12,13^. This combined with desirable features such as larger body size and ornaments, leads to selective harvesting of older males in many species, including the African savannah elephant^14^. However, there is no reason to believe there would be sex-based differences in the accumulation of information with age, and older males have the potential to occupy the same socio-cognitive role as females, particularly in species where all-male aggregations occur, such as the African savannah elephant.

Elephants possess detailed spatial knowledge of their core range, within which they travel using a “Euclidean-cognitive map”^15^. However, when navigating the periphery of their range, elephants switch to using habitual routes^15^. After prolonged use, these habitual routes can lead to “elephant pathways”, which are proposed to facilitate optimal foraging strategies by connecting predictable resources and landscape features such as drinking points^16^. Male elephants disperse from the natal herd between the age of 10–20 years and establish themselves in a separate bull society^17^. Males roam vast distances during their life time^18^, and social associations among males are weaker and more transitory than among females^19,20^. Between 25 and 30 years of age, males will experience their first stable ‘musth’, an annual cycle of temporary heightened reproductive state where males seek out females for mating^21^, with up to 74% of calves fathered by males in musth^22^. This temporal concentration of male sexual viability reduces intra-sexual conflict between males and opens the opportunity for male-male prosocial interactions among non-musth bulls^23^, including the opportunity for collective travel and information transfer from leaders to followers. Life expectancy including human-induced mortality has been estimated as 41 years for female African savannah elephants, but just 24 years for males^24^. Higher mortality could reflect the greater growth costs on adolescent males^25^, lower resistance to drought^26^, males’ greater involvement in wildlife conflict situations^27–29^, or the general risks associated with dispersal, such as lack of knowledge in navigating crucial resources in a new, complex and risky environment^30^. Thus, young adolescent males may gain considerable fitness benefits by associating with older males, with potentially decades more experience of utilising their environment safely and effectively, and older male elephants may act as repositories of ecological knowledge to younger males.

Here we quantify grouping behaviour and patterns of leadership in all-male elephant groups traveling on elephant pathways to and from the Boteti river in the Makgadikgadi Pans National Park (MPNP), Botswana. The MPNP is a “bull area”, with males representing 98% of elephant sightings^31^. We first quantify the extent to which male elephants of different age classes travel alone versus in all-male groups, showing that younger adolescent males show a preference for group travel. Second, we establish that mature adult bulls are more likely to lead by travelling at the front of all-male group processions. Finally, we compare leadership patterns between wet and dry seasons. Although there may be a heightened dependency on older males under particular ecological conditions (e.g. greater leadership by older knowledgeable males in the dry season when resources are scarcer^10^, or in the wet season when widely spread, unpredictable resources, such as timings of sprouting of vegetation, may require experienced knowledge to locate^32^), we find that leadership patterns by age were not influenced by the season.

## Results

1. Are adolescent male elephants less likely to be observed traveling alone?
  Lone travellers accounted for 20.8% of sightings on elephant pathways (n = 263/1,264). Adolescent males travelled alone significantly less often than predicted by chance, whilst mature adult bulls travelled alone significantly more often than predicted by chance (Permutation based likelihood ratio test of GLMM, χ^2^ (3) = 9.02^−7^, *p* < 0.001; Fig. 1).

**Figure 1:**
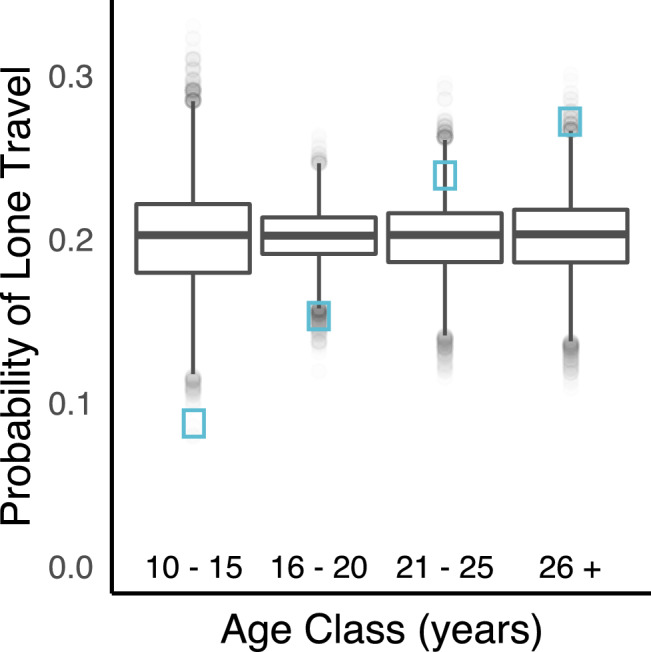
Adolescent males were less likely to travel alone than expected by chance. Observed probabilities of lone travel for the different age classes of male elephants (blue squares), plotted against permuted probabilities of lone travel (boxplots with median, interquartile range, minimum and maximum values). Observed probability for ages: 10–15 = 0.087, 95% CI random = (0.141–0.266), *p* = 0.001; 16–20 = 0.153, 95% CI random = (0.166–0.232), *p* = 0.010; 21–25 = 0.239, 95% CI random = (0.156–0.246), *p* = 0.105; 26+  = 0.272, 95% CI random = (0.154–0.248), *p* = 0.004.
2. Do older males lead social groups?
  Adolescents were significantly less likely than predicted by chance to travel at the front of groups and adult age classes were significantly more likely than predicted by chance to travel at the front of groups (Permutation based likelihood ratio test of GLMM, χ^2^ (3) = 7.83^−7^, *p* < 0.001; Fig. 2a). Adolescents were significantly more likely than predicted by chance to occupy middle positions in groups, and adult age classes significantly less likely (Permutation based likelihood ratio test of GLMM, χ^2^ (3) = 3.69^–10^, *p* < 0.001; Fig. 2b). No age class differed from random chance in their probability of being located at the rear of a traveling group (Permutation based likelihood ratio test of GLMM, χ^2^ (3) = 0.087, *p* = 0.185; Fig. 2c).

**Figure 2:**
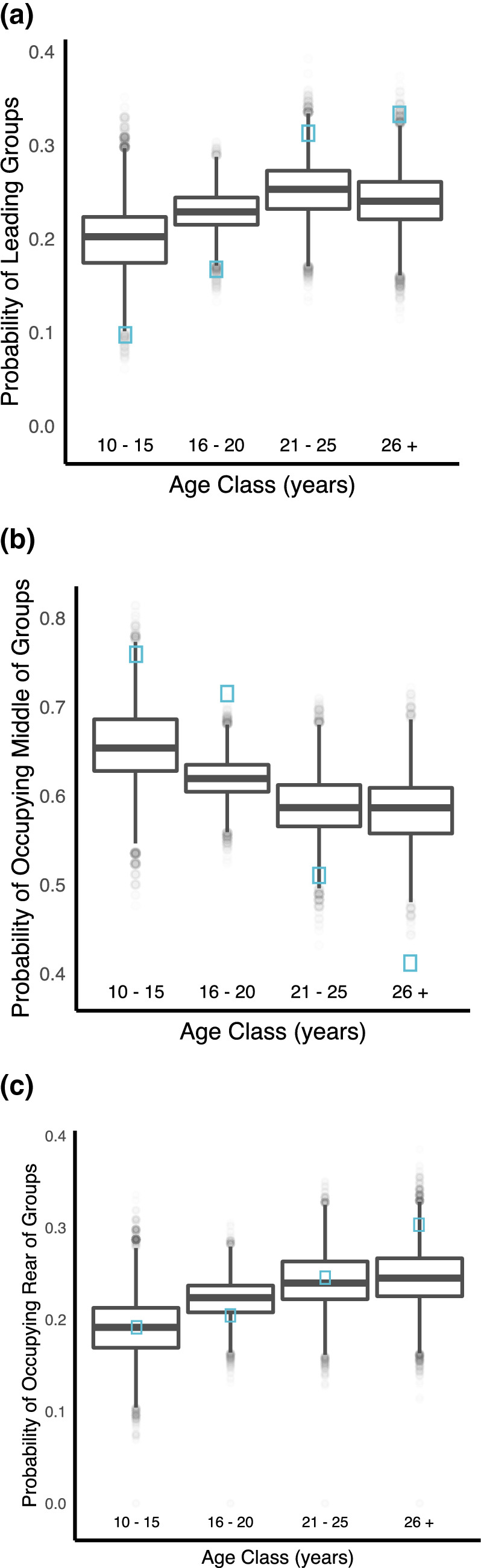
Probabilities of elephants occupying certain positions within all-male groups. Blue squares represent observed probabilities of occupying a certain position against box plots (with median, interquartile range, minimum and maximum values) of randomly permuted probabilities. (**a**) Older mature males were more likely to lead groups than expected by chance. Observed probability for ages: 10–15 = 0.097, 95% CI random = (0.128–0.277), *p* = 0.004; 16–20 = 0.167, 95% CI random = (0.187–0.270), *p* = 0.005; 21–25 = 0.313, 95% CI random = (0.194–0.312), *p* = 0.045; 26+  = 0.333, 95% CI random = (0.180–0.305), *p* = 0.004. (**b**) Adult males were less likely to occupy the middle position in traveling groups, and adolescents more likely than expected by chance. Observed probability for ages: 10–15 = 0.759, 95% CI random = (0.570–0.736), *p* = 0.012; 16–20 = 0.715, 95% CI random = (0.577–0.663), *p* < 0.001; 21–25 = 0.510, 95% CI random = (0.518–0.655), *p* = 0.028; 26+  = 0.412, 95% CI random = (0.511–0.654), *p* < 0.001. (**c**) Males of no age-class were more or less likely compared to chance to occupy the rear of traveling groups. All observed probabilities (blue squares) fell within range of randomly permuted probabilities of occupying the rear of groups (boxplots).
  Distance of the group from the river did not modify the tendency of any age class to hold the front (Permutation based likelihood ratio test of GLMM, Age Class*Distance: χ^2^ (3) = 0.664, *p* = 0.645), middle (Permutation based likelihood ratio test of GLMM, Age Class*Distance: χ^2^ (3) = 0.593, *p* = 0.591) or rear position of groups outside the range predicted by random chance (Permutation based likelihood ratio test of GLMM, Age Class*Distance: χ^2^ (3) = 0.417, *p* = 0.375).
3. Does season affect lone travel and leadership in male elephants?

There was no effect of season on tendency for males of different age classes to travel alone (Permutation based likelihood ratio test GLMM, Age Class*Season: χ^2^ (3) = 0.382, *p* = 0.383), nor was there an effect of season on the tendency of different age classes to lead groups (Permutation based likelihood ratio test of GLMM; Age Class*Season: χ^2^ (3) = 0.962, *p* = 0.965).

## Discussion

Our results highlight the importance of older mature males as leaders during collective movement in all-male groups of African savannah elephants. Adolescents travelled alone on elephant pathways significantly less than expected by chance, supporting our hypothesis that lone travel is riskier for younger, newly independent and less experienced individuals. In support of the hypothesis that mature adult bulls act as repositories for ecological knowledge, we found mature adult males were more likely to lead group movements, by occupying the front of all-male travelling groups. This age-related leadership pattern is consistent with findings in other species where older individuals have been shown to occupy leadership positions along migration routes, and during group-based foraging (whooping cranes, *Grus americana*^33^; resident killer whales, *Orcinus orca*^10^). Contrary to our prediction, leadership by mature adult bulls did not vary between the wet and dry season suggesting that mature bulls play a key role in the all-male elephant society, regardless of season.

**Figure 3:**
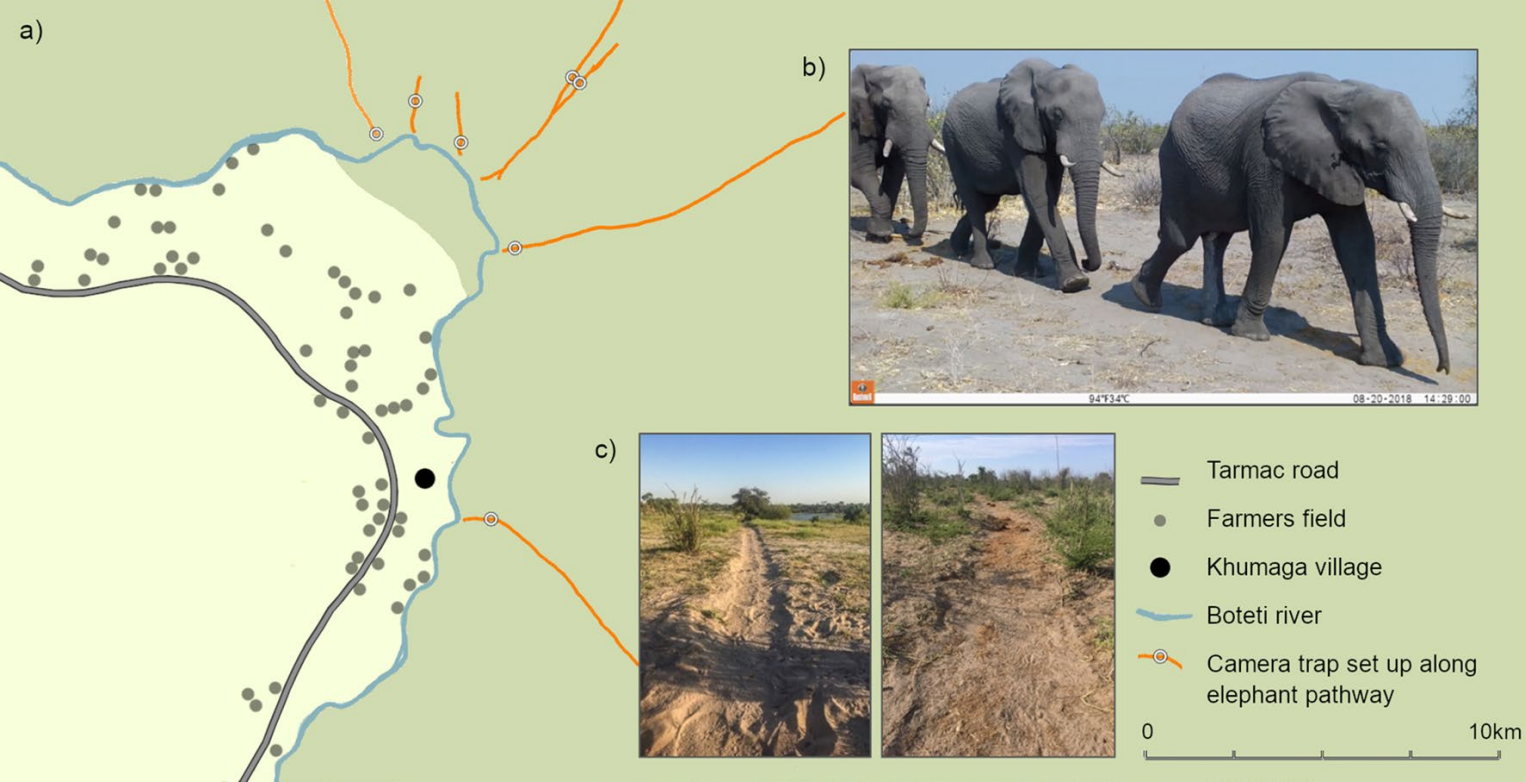
(**a**) Locations of sampled elephant pathways (orange lines) leading to the Boteti river during the course of the study. Dark green represents the MPNP protected area, and light green unprotected land, dominated by human activities, cattle and arable farming^28^. Aside from one functioning water hole, approximately 12 km northwest of the river, the river was the only permanent water source for wildlife in the area during this study. (**b**) Examples of images from camera traps (set to record video) of groups of elephants walking in a single file down the pathway. (**c**) Example images of elephant pathways in the MPNP.

In addition to enhanced experience, other mechanisms could lead to the observed age structuring of leadership patterns in this study. For example, leadership patterns could emerge due to a gradient of differences in walking speeds of different age classes, with adults walking faster than adolescents due to their larger size^34^. However, despite smaller absolute stride lengths in younger elephants, young African elephants can move as efficiently and as fast as older adults^35^, and non-musth bulls have been shown to decrease their average walking speed with age^36^. Furthermore, we found little evidence that size differences were driving our results. There was no observed size (age) gradient, with neither the smallest or largest bodied age class occupying the rear of groups more than chance. Predation risk is also known to differ depending on the spatial position of individuals in groups with those at the periphery being more vulnerable to predation^37^. Whilst a healthy adult bull has no natural predators, lions (*Panthera leo*) in Botswana’s Savute region predate young adolescent elephants at a 50% success rate and tend to attack elephants from the rear^38^. Adolescents did not avoid vulnerable rear positions of processions in our study, suggesting that predation risk is not a major factor in determining the position of animals in these traveling groups. Furthermore, closer to the river, there is an increased threat of predation from lions^39^ and of human encounter (Fig. 3a) both of which can affect male mortality, with human activity being the leading cause of adult male mortality in many populations^24,40,41^. However, we found no effect of distance from the river on traveling positions, further suggesting that such mortality risks are not driving the observed leadership patterns.

Leadership patterns can also emerge in animal groups due to variation among individuals in motivational state^42^ with individuals most incentivised to change the environment or attain a goal often arising as leaders (e.g. food deprived fish leading shoals^43^, lactating zebra mares, *Equus burchellii*, leading groups to water^44^). Various studies suggest that smaller bodied adolescents have greater drinking needs^45^, and require higher quality forage relative to older males^46,47^; suggesting that younger adolescents should be the most motivated to travel at the front of groups in order to reach foraging and drinking locations connected by pathways. Such patterns were not observed in the current study, suggesting internal condition was not the key determinant of which individuals arose as leaders in groups.

Our finding that mature bulls held leading positions at the front of groups, and that no age class occupied the rear more than chance is in stark contrast to matriarchs in breeding herds of African savannah elephants, who have been observed to initiate group movements and indicate the direction of travel before retreating to the back positions of groups^48^. This may point to the different motivations behind group-travel and different follower-leader dynamics in all-male groups compared to breeding herds. Whilst male African elephants are “atomistic” in their community, with each individual male representing a unit that can choose to break off or join with other males based on current pressures and motivations; females in African elephant breeding herd groups are “molecular”^49^, each unit consisting of a tightly bonded stable family group, led by a matriarch that can join and break off with other families in increasing levels of social organisation^50,51^. Whilst elephants in all-male groups are flexible to break up or fuse based on immediate, individual needs, female family groups are principally held together by inclusive fitness and kin selection^19^. A matriarch is therefore more likely to benefit from monitoring group members and their safety^52^ compared to any individual male. If matriarchs benefit by actively maintaining their followers, traveling at the rear of groups where family members can be monitored, may be more effective than traveling at the front^48^. In contrast, in older male elephants leadership may be a far more passive process, with older individuals making choices (based on enhanced experience) and being tolerant to the active followers that target and trail behind them^4,53^.

Across their geographic range, mature bulls represent a minority in the population^24^. Exacerbating this, older bull elephants are overwhelmingly targeted in both illegal poaching activity^41^ and legal trophy hunting activity^14^, because of their larger body size and increased trophy size (ivory tusks) with age^54,55^. Only male elephants are targeted for trophy hunting, and operators often put minimum ages, corresponding with greater tusk weights, on targeted animals^56–58^. Poaching, conflict with communities, and hunting activity are the lead causes of mortality in mature bulls, and their numbers are declining at a rapid rate^40^. Trophy hunting divides conservationists for its potential benefits and negative impacts^59^. Supporters argue when a quota system for trophies is managed carefully following ecological theory, trophy hunting only removes a few older males with low reproductive value from a population, which should have a negligible effect on the wider environment^12,13^. However, researchers frequently voice concern that this model fails to consider the mating and social system of the species of interest when applied to African savannah elephants^17,60,61^. Our finding that mature adult bulls act as leaders during collective movement complements existing research that highlights the central role of mature bulls in all-male African savannah elephant societies. For example, older bulls are most commonly targeted as nearest neighbours by males of all ages^62^, and have a greater number of associates than younger males^20^. Removal of older mature bulls not only removes the prime breeders (the oldest individuals in the population sire the most offspring^21,22,63^), but, as our study suggests, it also removes individuals with a central role in the male society, particularly in the context of their role in leading younger naïve males between critical resources.

At the time of writing, Botswana had recently announced a decision to recommence elephant trophy hunting, and has been issued export quotas for tusks from trophies of 400 elephants by CITES for 2020^64^. An article supporting a return to trophy hunting in Botswana^65^ reported of an effective quota system that “is regulated such that only old male animals were killed”. We argue that such selective hunting would not be sustainable, and removal of older mature bulls from the population could disrupt the wider bull society and the inter-generational flow of information concerning decades of accumulated ecological knowledge, such as effective navigation and location of critical resources. We argue mature bulls occupy a similar role in male elephant society as old female matriarchs in breeding herds^8,9^ and require equal protection.

## Methods

Elephants travelling along elephant pathways towards and away from the Boteti river (MPNP, Botswana, Fig. 3a) were observed between October 2017 and September 2018. The MPNP is as a “bull area”, with males representing 98% of elephant sightings^31^. Male predominance can potentially be attributed to the MPNP being at the fringes of the African savannah elephants’ range in Botswana^66^, and the region has received increasing numbers of male elephants since the returning flow of the Boteti River in 2009 following a 19-year dry phase^31^. Male elephants are non-territorial and the male population of the MPNP is largely transitory, with individual bulls staying on average 47 days in the area^67^.

To record group travel, we positioned camera traps (2017 Bushnell Aggressor HD No-glow, set to record video, Supplementary S1 & S2) along seven elephant pathways (Fig. 3a). Due to the large volume of video collected, analysis was conducted on a subset of footage from the last seven sampled days of every sampled month (elephant sightings n = 1,264). Within this data set, a human observer used distinguishing features such as ear notches, tears and holes, tusk length, girth and morphology, skin wrinkles, tail length and other abnormalities to identify individuals (n individuals = 1,097). Validation of the identification methods showed that elephants could be correctly identified with a 100% success rate (Supplementary S3). We used a combination of characteristics such as body size and shoulder height^25^, head morphology and size, and tusk girth and splay^54^ to assign age class to individuals. Males were categorised as young adolescents, 10–15 years (n = 150), older adolescents, 16–20 years (n = 487), young adults, 21–25 years (n = 252) and mature adults 26 + years (n = 208). Recent research in the MPNP found a similar age demographic^28^. Analysis based on finer-scale categorisation of older bulls by age that is typical for male elephants in other study areas across Africa (26–35 years & 36 years+^62^; 25–34.9 years, 35–49.9 years & 50+^68^) was not possible in our study due to a small sample size of individuals older than 36 years (n = 18). We therefore set our older bull category as 26 years + , and argue this is an appropriate categorisation because (i) it represents the age where males begin exhibiting sexually active periods (musth) and experiencing alterations to sexual and social interests and behaviours^63^, and (ii) male elephants over this age are the prime targets of trophy hunting and poaching activity due to the weight of their ivory^41,56–58^.

Elephants walked in single file along elephant pathways, making order of travel easy to quantify (Fig. 3b). Existing studies use successful initiation of group movements, as well as positioning within group to indicate leadership in collective movements^10,34,48,69^. Lacking information concerning initiations, we used the latter definition, assigning leadership to those at the front of single file processions. In processions of African savannah elephants breeding herds, matriarchs are more likely to occupy the back of travelling groups, suggesting leadership from the rear^48^. We also therefore analysed age structure of elephants occupying middle and rear positions in all-male groups separately. Group assignment was determined based on the time that an individual passed the camera trap in relation to the previous passing elephant moving in the same direction. The majority of following events occurred within 10 min (Supplementary Figure S1a), we therefore set an eleven-minute difference to the previous elephant to pass as the cut-off period to indicate the start of a new group. A large number of following events occurred with a 0 or 1 min time stamp difference (Supplementary Figure S1b), we therefore further explored leadership patterns in these smaller subgroups and found qualitatively similar patterns to our main analysis (Supplementary S4 for more detailed rationale behind grouping assignment and additional subgroup analyses).

Patterns of male elephant grouping were analysed in R using generalized logistic mixed-effects models (GLMMs), with statistical significance assessed using permutation-based null models. We first investigated if males of adult age classes were more likely to travel alone (as opposed to in all-male groups), and adolescent age classes less likely than predicted by chance. We fit a GLMM with a binomial error structure and a logit link function, predicting lone travel (dependent variable) by age class (independent variable), controlling for elephant ID as a random effect. We compared the estimates from this model to those generated from 20,000 permutations of the data in which we randomly re-assigned individuals to groups within the same season, maintaining the total number of times each individual was seen and the size of each group. A permutation approach allowed us to control for patterns in the data set owing to its inherent structure, including having a greater number of adolescents present in the population.

To test the prediction that mature bulls led all-male groups more than predicted by chance, groups containing at least 1 adult and 1 adolescent (n individuals = 725, n groups = 182) were assessed for the position of travel of individuals. Binomial GLMMs were fit, and the estimates obtained for the observed data set were compared to 20,000 randomised data sets, with age composition of each group maintained, whilst position of individuals within groups were randomly shuffled in each permutation. Separate models were run predicting tendency to occupy front, middle and rear positions in groups (dependent variables) by age class (independent variable), again controlling for elephant ID as a random effect. Ages of elephants traveling in the middle of groups was assessed in all-male groups with a group size of at least 3 with at least 1 adult and 1 adolescent (n individuals = 631, n groups = 132).

The Boteti region has the greatest reported level of human-wildlife conflict in Botswana^70^. Both lion^39^ and human conflict risk becomes higher for elephants closer to the Boteti river (Fig. 3a). Such threats may make the risk of being at both the front and rear of groups greater for vulnerable individuals^37^, which may affect patterns of leadership. To test for these effects, we tested if distance of camera set up from the river modified elephants of different age classes tendency to hold certain position within groups, by including distance as an interaction term in the above models. We similarly analysed any interaction of age class with season (wet vs. dry) on models of lone travel and leadership, to explore whether there was variation in the tendency for mature bulls to act as leaders between seasons. We used daily rainfall records to determine onset of wet and dry seasons (Supplementary S5).

Mean size of traveling groups did not differ between pathways, and the inclusion of pathway location as a random effect did not qualitatively alter the outputs of models (Supplementary S6 & S7). Data from the different pathways were therefore pooled for analyses.

Musth bulls were identified by a combination of heavy temporal gland secretion, urine dribbling, and green staining around the penis^21^. Musth bulls represented a small number of elephants observed in the study (n = 19). However, because musth affects only adult age classes, we reran all permutation based GLMM’s removing musth bulls from the dataset and found qualitatively similar results in all models (Supplementary S8). Musth males do not therefore appear to be driving our results.

### Ethics standards

This work received approval from the University of Exeter Research Ethics Committee (application ID: eCLESPsy000545v3.2).

## Supplementary information

Supplementary Information.

## Data Availability

Due to the sensitive nature of reporting on elephant locations and numbers, the data that support the findings of this study are available on reasonable request from the corresponding author.

## Code availability

R code for running permutation based generalized logistic mixed-effects models available upon request from the corresponding author [C.A.].
