# Supplementary material for: Importance of old bulls: leaders and followers in collective movements of all-male groups in African savannah elephants (Loxodonta africana)
Source: Sci Rep. 2020 Sep 3;10:13996. doi: 10.1038/s41598-020-70682-y (PMC7471917; doi:10.1038/s41598-020-70682-y)
Supplement: Supplementary file 1 — Supplementary Information. [file 41598_2020_70682_MOESM1_ESM.docx]

**Supplementary Information**

**Importance of Old Bulls: Leaders and Followers in Collective Movements of All-Male Groups in African Savannah Elephants (*Loxodonta africana*)**

**Connie RB Allen1, Lauren JN Brent1, Thatayaone Motsentwa2, Michael N Weiss1 and Darren P Croft1**

1 Centre for Research in Animal Behaviour, College of Life and Environmental Sciences, University of Exeter, Exeter EX4 4QG, UK.

2 Elephants for Africa, UK address: 5 Balfour Road, London N5 2HB, UK. Botswana address: Mailbox HAK 148 HAKb, Maun, Botswana.

Correspondence to [connierballen@gmail.com](mailto:connierballen@gmail.com) (Connie RB Allen)

**Supplementary S1: Description of identification of elephant pathways and camera trap set up.**

Pathways measured on average 84.8 cm in width (SD=6 cm, n pathways=7, n measurements=46, range n measurements per pathway=5-16), with measurements taken at random points along the pathway within a safe 50m distance of a vehicle. Pathways were devoid of vegetation, fully blanketed at points with old, dried flattened dung, and maintained by repeated single file movement of elephants. To ensure uniformity of camera trigger response, and to ensure as much of the animal was captured in the frame as possible, we placed cameras on 2m high gum poles positioned 9m, at an angle of 45°, either side of a central point on the pathway. By placing a camera facing inwards on either side of the focal point, head on footage (essential for aging purposes) was available for both elephants walking towards and away from the river. Cameras were active for a cumulative 8942 study hours.

**Supplementary S2: Table S1: A total of 25 mammal species were also observed to utilise elephant pathways.**

| **Species** | **Total on Camera Traps** | **Total on Main Pathway** | **% on Main Pathway** |
| --- | --- | --- | --- |
| Elephant  *Loxodonta africana* | 3858 | 3365 | 87 |
| Zebra  *Equus quagga* | 6787 | 875 | 13 |
| Wildebeest  *Connochaetes taurinus* | 802 | 117 | 15 |
| Giraffe  *Giraffa giraffa* | 118 | 65 | 55 |
| Jackal  *Canis mesomelas* | 89 | 65 | 73 |
| Porcupine  *Hystrix africaeaustralis* | 42 | 40 | 95 |
| Lion  *Panthera leo* | 25 | 24 | 96 |
| Steinbok  *Raphicerus campestris* | 61 | 23 | 38 |
| Brown Hyena  *Hyaena brunnea* | 28 | 23 | 82 |
| Kudu  *Tragelaphus strepsiceros* | 96 | 19 | 20 |
| Cape Fox  *Vulpus chama* | 14 | 10 | 71 |
| Impala  *Aepyceros melampus* | 16 | 7 | 44 |
| Spotted Hyena  *Crocuta crocuta* | 6 | 6 | 100 |
| Leopard  *Panthera pardus* | 6 | 5 | 83 |
| Honey Badger  *Mellivora capensis* | 5 | 5 | 100 |
| Common Duiker  *Sylvicapra grimmia* | 8 | 4 | 50 |
| Bat Eared Fox  *Otocyon megalotis* | 4 | 3 | 75 |
| Wild Cat  *Felis lybica* | 4 | 1 | 25 |
| Caracal  *Caracal caracal* | 1 | 1 | 100 |
| Hippo  *Hippopotamus amphibius* | 1 | 1 | 100 |
| Serval  *Leptailurus serval* | 1 | 1 | 100 |
| White Rhino  *Ceratotherium simum* | 1 | 1 | 100 |
| Cattle  *Bos taurus* | 84 | 0 | 0 |
| Zorilla  *Ictonyx striatus* | 3 | 0 | 0 |
| Gemsbok  *Oryx gazella* | 1 | 0 | 0 |

**Supplementary S3: Validation of Elephant ID’s**

All individuals were uniquely identified by a human observer using distinguishing features such as ear notches, tears and holes, tusk length, girth and morphology, skin wrinkles, tail length and hair fullness, and other abnormalities (n individual elephants = 1097). Reliability of identification was validated by presenting footage of anonymised elephants to a blind researcher. Using characteristics listed above, the researcher assigned the elephant to an ID in the data base. 100% of elephants were assigned their correct ID number (n = 30).

**Supplementary S4: Assignment of group membership**

Previous studies have defined elephant groups as individuals within 100m of each other, coordinated in their activities [1,2]. Such large distances between coordinated individuals can be achieved due to the species’ exceptional long-distance chemical, seismic and infrasonic communication abilities [3,4]. Furthermore, the sparsely vegetated open *Acacia* Savannah-type habitat of the MPNP study area [5] makes for good visual range between individuals walking on pathways.

To assign group membership we plotted the time stamp differences of individual elephants from the previous individual to pass the camera trap on the pathway, traveling in the same direction, to discern appropriate cut-off times for group membership. The majority of following events occurred within 10 minutes, we therefore set an eleven-minute difference to the previous elephant to pass as the cut-off period to indicate the start of a new group (Figure S1). Using previous research we estimate this 10 min cut-off period will translate to an inter-individual distance of between 64 meters (if assuming average walking speed of elephants in protected areas [6]) and 201 meters (if assuming faster walking speed of elephants in corridors [6]), a reasonable distance over which elephants will be able to maintain a visual line of sight in the habitat and communicate.

Time stamp between consecutive elephants (min +1)


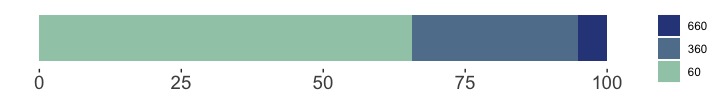


Time stamp difference to previous group member (min)

Figures 6 & 7 (right):

0-1

2-5

6-10

Percentage of Elephants (%)

Figures 6 & 7 (right):


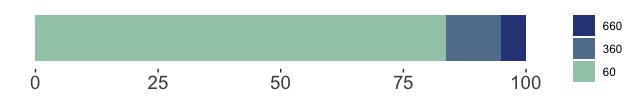

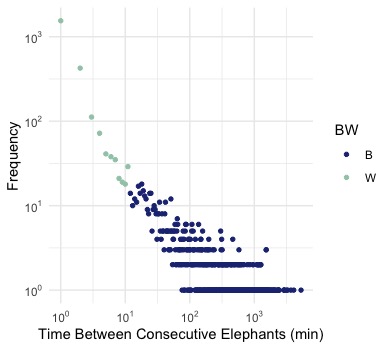

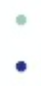


Grouping Assignment

Same group

Different group

a)

b)

Frequency

Figures 6 & 7 (right):

Figure S1 a) Histogram (plotted on logarithmic scale) of the time difference between pairs of consecutive observations of elephants travelling on pathways. Groups were defined as individuals detected on the same pathway, moving in the same direction with a 10 minute or less time stamp to the previous passing individual. A time stamp difference of +1 was added to all values to allow visualisation of 0-minute time stamp difference on a logarithmic scale. For between group recordings, only 10.21% of groups were separated by a 11-20 minutes time stamp, with >20 minute differences separating groups in the majority of cases (range: 20-1437 minutes) b) Percentage of elephants observed within certain time brackets of their previous group member.

Considering that within these defined groups, 83.65% of following events occurred with a time stamp difference of 0 or 1 minute to their previous group member (Figure S1b), we reran our main models of lone travel and position held within groups, to determine if the same patterns would be observed considering only smaller tightly bunched “subgroups” of male elephants, to affirm confidence in our key hypotheses of older bulls leading, and younger bulls following in all-male groups. We found qualitatively similar results in all models (Tables S2-S5, Figures S2-S5), albeit an increase in probability of lone travel in all ages, and inevitable increases in probabilities for holding certain positions in groups due to smaller group sizes.

Table S2: Likelihood ratio test of permutation-based GLMM, male elephant lone travel by age class, elephant ID included as random effect, 1-minute cut-off to group membership.

| Df | AIC | LRT | Pr (Chi) | Randomised P |
| --- | --- | --- | --- | --- |
| 3 | 1553.458 | 42.903 | 2.580 e-09 | **5.000 e-05 *** |


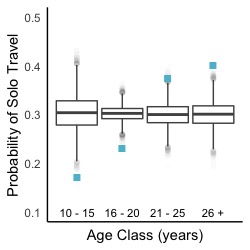


Figure S2: Lone travel in different age classes with 1-minute cut-off applied to group membership. Adolescent males were less likely to travel alone than expected by chance, and adults more likely. Observed probabilities of lone travel for the different age classes of male elephants (blue squares), plotted against permuted probabilities of lone travel (boxplots with median, interquartile range, minimum and maximum values). Observed probability for ages: 10-15 = 0.177, 95% CI random = (0.235-0.373), p < 0.001; 16-20 = 0.236, 95% CI random = (0.272-0.332), p < 0.001; 21-25 = 0.379, 95% CI random = (0.253-0.347), p = 0.001; 26+ = 0.406, 95% CI random = (0.252-0.350), p < 0.001.

Table S3: Likelihood ratio test of permutation-based GLMM, male elephant leadership of all-male groups by age class, elephant ID included as random effect, 1-minute cut-off to group membership.

| Df | AIC | LRT | Pr (Chi) | Randomised P |
| --- | --- | --- | --- | --- |
| 3 | 651.669 | 40.241 | 9.473 e-09 | **5.000 e-05 *** |


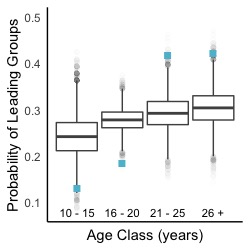


Figure S3: Leadership of all-male groups in different age classes with 1-minute cut-off applied to group membership. Adolescent males were less likely to lead groups than expected by chance, and adults more likely, with highest probabilities in the oldest age class. Observed probabilities of leadership for the different age classes of male elephants (blue squares), plotted against permuted probabilities of leadership (boxplots with median, interquartile range, minimum and maximum values). Observed probability for ages: 10-15 = 0.136, 95% CI random = (0.152-0.344), p = 0.024; 16-20 = 0.190, 95% CI random = (0.229-0.326), p = 0.001; 21-25 = 0.423, 95% CI random = (0.220-0.372), p = 0.001; 26+ = 0.426, 95% CI random = (0.226-0.383), p = 0.004.

Table S4: Likelihood ratio test of permutation-based GLMM, male elephant middle travel in all-male groups by age class, elephant ID included as random effect, 1-minute cut-off to group membership.

| Df | AIC | LRT | Pr (Chi) | Randomised P |
| --- | --- | --- | --- | --- |
| 3 | 597.761 | 30.673 | 9.963 e-07 | **1.000 e-04 *** |

**
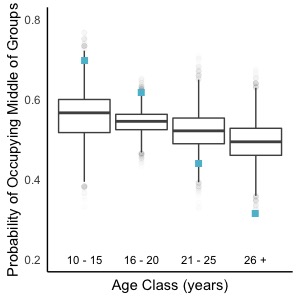
**

Figure S4: Middle travel in all-male groups in different age classes with 1-minute cut-off applied to group membership. Adolescent males were more likely to occupy the middle of groups than expected by chance, and the oldest age class of adults less likely. Observed probabilities of occupying the middle of groups for the different age classes of male elephants (blue squares), plotted against permuted probabilities (boxplots with median, interquartile range, minimum and maximum values). Observed probability for ages: 10-15 = 0.704, 95% CI random = (0.450-0.669), p = 0.010; 16-20 = 0.623, 95% CI random = (0.492-0.600), p = 0.004; 21-25 = 0.446, 95% CI random = (0.436-0.617), p = 0.075; 26+ = 0.322, 95% CI random = (0.404-0.584), p < 0.001.

Table S5: Likelihood ratio test of permutation-based GLMM, male elephant rear travel in all-male groups by age class, elephant ID included as random effect, 1-minute cut-off to group membership.

| Df | AIC | LRT | Pr (Chi) | Randomised P |
| --- | --- | --- | --- | --- |
| 3 | 654.607 | 4.479 | 0.214 | 0.378 |


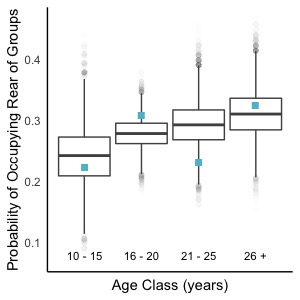


Figure S5: Rear travel in all-male groups in different age classes with 1-minute cut-off applied to group membership. Males of no age-class were more or less likely compared to chance to occupy the rear of traveling groups. All observed probabilities (blue squares) fell within range of randomly permuted probabilities of occupying the rear of groups (boxplots). Observed probability for ages: 10-15 = 0.227, 95% CI random = (0.149-0.333), p = 0.825; 16-20 = 0.312, 95% CI random = (0.231-0.329), p = 0.193; 21-25 = 0.236, 95% CI random = (0.220-0.369), p = 0.118; 26+ = 0.328, 95% CI random = (0.237-0.388), p = 0.671.

**Supplementary S5: Determination of Season**

Onset of the wet and dry season in each year was determined using daily rainfall measured at three locations in the study area (GPS coordinates: i. S: 20°27'28.67", E: 24°30'58.66" ii. S: 20°28'19.94", E: 24°31'3.46 iii. S: 20°12'50.94", E: 24°35'40.53"). Onset of the wet season was determined by the first rainfall over 15 mm, as in previous years this volume tended to signal the beginning of regular rainfall. The onset of the dry season was defined as 14 days after the last rainfall, the lag was to account for potential presence of surface water away from the river, and the period following the last rains where vegetation was still of high quality.

**Supplementary S6: Summary statistics for average group size between sampled pathways.**

Table S6: Summary statistics from the 7 sampled pathways (n elephant sightings=1316, n groups=548). There was no significant difference between pathways for average group size of all-male groups observed on camera traps, lone travellers included (Kruskal Wallis χ2(6) = 9.445, p = 0.150).

| **Pathway N.** | **N. Groups** | **Mean Group Size** | **SD** | **Median** | **IQR** |
| --- | --- | --- | --- | --- | --- |
| 1 | 179 | 2.58 | 2.22 | 2 | 2 |
| 2 | 31 | 2.32 | 2.14 | 1 | 2 |
| 3 | 110 | 2.39 | 2.88 | 1 | 1 |
| 4 | 144 | 2.28 | 1.70 | 2 | 2 |
| 5 | 47 | 2.36 | 1.47 | 2 | 2 |
| 6 | 22 | 2.32 | 1.91 | 2 | 2 |
| 7 | 15 | 1.87 | 0.92 | 2 | 2 |

Table S7: Summary statistics from the 7 sampled pathways, excluding lone male travellers (n elephant sightings=1052, n groups=284). There was no significant difference between pathways for average group size of all-male groups observed on camera traps, lone travellers excluded (Kruskal Wallis χ2(6) = 4.064, p = 0.668).

| **Pathway N.** | **N. Groups** | **Mean Group Size** | **SD** | **Median** | **IQR** |
| --- | --- | --- | --- | --- | --- |
| 1 | 103 | 3.75 | 2.32 | 3 | 2 |
| 2 | 14 | 3.93 | 2.34 | 3 | 2.5 |
| 3 | 41 | 4.73 | 3.69 | 3 | 4 |
| 4 | 77 | 3.40 | 1.64 | 3 | 2 |
| 5 | 29 | 3.21 | 1.26 | 3 | 2 |
| 6 | 12 | 3.42 | 2.02 | 3 | 2 |
| 7 | 8 | 2.62 | 0.52 | 3 | 1 |


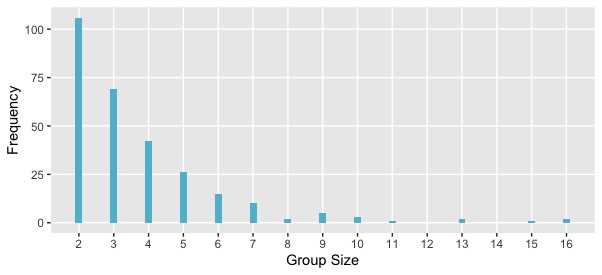
Figure S6: Histogram of observed all-male group sizes on pathways, lone males excluded.

**Supplementary S7: Effect of pathway location on permutation-based generalized logistic mixed-effects models (GLMMs) of lone travel and leadership of all-male groups.**

To control for the effect of pathway location on our observed results, we reran models of lone travel and leadership, modelling pathway location as a random effect in permutation-based generalized logistic mixed-effects models. There were no changes to overall model or individual coefficient significances when including pathway location as a random effect, except that the tendency for 21-25 year olds to be alone more than predicted by chance was significant. Overall, we could therefore be confident that sample location site was not a significant constraint on the results we observed.

1. Likelihood ratio test, coefficients and probabilities of permutation-based GLMM, male elephant lone travel by age class, pathway location included as random effect.
2. LRT table

| Df | AIC | LRT | Pr (Chi) | Randomised P |
| --- | --- | --- | --- | --- |
| 3 | 1295.103 | 30.625 | 1.019 e-06 | **5.000 e-05 *** |

| Age class (years) | Coefficient estimate | Std. Error | Z value | Pr(>\|z\|) | Randomised P |
| --- | --- | --- | --- | --- | --- |
| 10 | -2.177 | 0.273 | -7.961 | 1.710 e-15 | **1.000 e-04 *** |
| 16 | -1.557 | 0.134 | -11.660 | 2.036 e-31 | **0.008 *** |
| 21 | -1.043 | 0.150 | -6.959 | 3.420 e-12 | **0.009 *** |
| 26 | -0.903 | 0.153 | -5.888 | 3.915 e-09 | **0.001 *** |

1. Coefficients
2. Probabilities

| Age class (years) | Probability of solo travel |
| --- | --- |
| 10 – 15 | 0.102 |
| 16 – 20 | 0.174 |
| 21 – 25 | 0.261 |
| 26+ | 0.289 |

1. Likelihood ratio test, coefficients and probabilities of permutation-based GLMM, male leadership of all-male groups, pathway location included as random effect.
2. LRT table

| Df | AIC | LRT | Pr (Chi) | Randomised P |
| --- | --- | --- | --- | --- |
| 3 | 800.454 | 31.829 | 5.686 e-07 | **5.000 e-05 *** |

1. Coefficients

| Age class (years) | Coefficient estimate | Std. Error | Z value | Pr(>\|z\|) | Randomised P |
| --- | --- | --- | --- | --- | --- |
| 10 | -2.122 | 0.338 | -6.280 | 3.390 e-10 | **0.002 *** |
| 16 | -1.520 | 0.154 | -9.868 | 5.707 e-23 | **0.003 *** |
| 21 | -0.734 | 0.169 | -4.347 | 1.380 e-05 | **0.034 *** |
| 26 | -0.644 | 0.174 | -3.705 | 2.117 e-04 | **0.003 *** |

1. Probabilities

| Age class (years) | Probability of leading all-male groups |
| --- | --- |
| 10 – 15 | 0.107 |
| 16 – 20 | 0.179 |
| 21 – 25 | 0.324 |
| 26+ | 0.344 |

**Supplementary S8: Musth excluded permutation-based likelihood ratio tests and coefficients of GLMMs.**

Musth bulls were included for analysis of group orders and lone travel. 19 bulls in the data set were observed to be in musth, 15 moving as lone travellers, and 4 in all-male groups. Because musth state affects only adult age classes, and because there were significantly more musth bulls in the wet season (2.5% of elephants) compared to dry season (0.9% of elephants) (Chi Square Goodness of Fit: X*2* (1) = 35.9, *p* < 0.001), we also evaluated models in which musth bulls were removed. None of our models demonstrated qualitative differences when musth bulls were excluded from the data set. Musth males do not therefore appear to be driving our results. Elephant ID included as a random effect in all models.

1. Likelihood ratio test and coefficients of permutation-based GLMM, male elephant lone travel by age class, musth excluded model.
2. LRT table

| Df | AIC | LRT | Pr (Chi) | Randomised P |
| --- | --- | --- | --- | --- |
| 3 | 1246.989 | 23.335 | 3.438 e-05 | **0.001 *** |

1. Coefficients

| Age class (years) | Coefficient estimate | Std. Error | Z value | Pr(>\|z\|) | Randomised P |
| --- | --- | --- | --- | --- | --- |
| 10 | -2.290 | 0.327 | -6.994 | 2.669 e-12 | **0.009 *** |
| 16 | -1.662 | 0.203 | -8.174 | 2.979 e-16 | **0.030 *** |
| 21 | -1.156 | 0.189 | -6.123 | 9.176 e-10 | 0.109 |
| 26 | -1.122 | 0.182 | -6.180 | 6.412 e-10 | **0.029 *** |

1. Likelihood ratio test and coefficients of permutation-based GLMM, male leadership of all-male groups, musth excluded model
2. LRT table

| Df | AIC | LRT | Pr (Chi) | Randomised P |
| --- | --- | --- | --- | --- |
| 3 | 782.603 | 29.833 | 1.496 e-06 | **5.000 e-05 *** |

1. Coefficients

| Age class (years) | Coefficient estimate | Std. Error | Z value | Pr(>\|z\|) | Randomised P |
| --- | --- | --- | --- | --- | --- |
| 10 | -2.163 | 0.383 | -5.648 | 1.627 e-08 | **0.006 *** |
| 16 | -1.582 | 0.213 | -7.438 | 1.024 e-13 | **0.006 *** |
| 21 | -0.780 | 0.195 | -4.003 | 6.251 e-05 | **0.046 *** |
| 26 | -0.670 | 0.197 | -3.408 | 6.547 e-04 | **0.002 *** |

1. Likelihood ratio test and coefficients of permutation-based GLMM, male middle travel in all male groups, musth excluded model
2. LRT table

| Df | AIC | LRT | Pr (Chi) | Randomised P |
| --- | --- | --- | --- | --- |
| 3 | 828.093 | 45.922 | 5.894 e-10 | **5.000 e-05 *** |

1. Coefficients

| Age class (years) | Coefficient estimate | Std. Error | Z value | Pr(>\|z\|) | Randomised P |
| --- | --- | --- | --- | --- | --- |
| 10 | 1.088 | 0.266 | 4.090 | 4.313 e-05 | **0.020 *** |
| 16 | 0.923 | 0.153 | 6.041 | 1.529 e-09 | **1.000 e-04 *** |
| 21 | 0.029 | 0.172 | 0.168 | 8.667 e-01 | **0.030 *** |
| 26 | -0.385 | 0.186 | -2.066 | 3.886 e-02 | **1.000 e-04 *** |

1. Likelihood ratio test and coefficients of permutation-based GLMM, male rear travel in all male groups, musth excluded model
2. LRT table

| Df | AIC | LRT | Pr (Chi) | Randomised P |
| --- | --- | --- | --- | --- |
| 3 | 778.069 | 6.407 | 0.093 | 0.176 |

1. Coefficients

| Age class (years) | Coefficient estimate | Std. Error | Z value | Pr(>\|z\|) | Randomised P |
| --- | --- | --- | --- | --- | --- |
| 10 | -1.386 | 0.264 | -5.260 | 1.437 e-07 | 0.881 |
| 16 | -1.366 | 0.143 | -9.520 | 1.736 e-21 | 0.289 |
| 21 | -1.099 | 0.178 | -6.166 | 7.007 e-10 | 0.811 |
| 26 | -0.825 | 0.177 | -4.668 | 3.045 e-06 | 0.087 |

1. Likelihood ratio tests of permutation-based GLMMs, camera trap setup distance from rivers’ effect on different age-classes position of travel in groups, musth excluded models.
2. LRT table effect of distance on leadership of all-male groups, musth excluded model

| Df | AIC | LRT | Pr (Chi) | Randomised P |
| --- | --- | --- | --- | --- |
| 3 (Distance*AgeClass) | 760.737 | 2.050 | 0.562 | 0.559 |

1. LRT table effect of distance on middle travel in all-male groups, musth excluded model

| Df | AIC | LRT | Pr (Chi) | Randomised P |
| --- | --- | --- | --- | --- |
| 3 (Distance*AgeClass) | 789.868 | 2.381 | 0.497 | 0.479 |

1. LRT table effect of distance on rear travel in all-male groups, musth excluded model

| Df | AIC | LRT | Pr (Chi) | Randomised P |
| --- | --- | --- | --- | --- |
| 3 (Distance*AgeClass) | 778.941 | 2.623 | 0.453 | 0.407 |

1. Likelihood ratio test of permutation-based GLMM, effect of season on lone travel by different age classes, musth excluded models

| Df | AIC | LRT | Pr (Chi) | Randomised P |
| --- | --- | --- | --- | --- |
| 3 (Season*AgeClass) | 1224.294 | 2.688 | 0.442 | 0.445 |

1. Likelihood ratio test of permutation-based GLMM, effect of season on leadership of all-male groups by different age classes, musth excluded models

| Df | AIC | LRT | Pr (Chi) | Randomised P |
| --- | --- | --- | --- | --- |
| 3 (Season*AgeClass) | 753.381 | 0.124 | 0.989 | 0.991 |

**Cited Literature**

[1] Wittemyer, G., Douglas-Hamilton, I. & Getz, W. M. The socioecology of elephants: analysis of the processes creating multitiered social structures. Animal Behaviour 69, 1357–1371 (2005).

[2] Murphy, D., Mumby, H. S. & Henley, M. D. Age differences in the temporal stability of a male African elephant (Loxodonta africana) social network. Behavioral Ecology (2019) doi:10.1093/beheco/arz152.

[3] Langbauer, W. R. Elephant communication. Zoo Biology 19, 425–445 (2000).

[4] O’Connell-Rodwell, C. E. Keeping an “Ear” to the Ground: Seismic Communication in Elephants. Physiology 22, 287–294 (2007).

[5] Brooks, C.J. The foraging behaviour of Burchell's zebra (Equus burchelli antiquorum). PhD Thesis, (University of Bristol, 2005).

[6] Douglas-Hamilton, I., Krink, T. & Vollrath, F. Movements and corridors of African elephants in relation to protected areas. Naturwissenschaften 92, 158–163 (2005).
